# P-360. Impact of a tiered masking intervention on healthcare worker illness and absenteeism during respiratory viral season

**DOI:** 10.1093/ofid/ofae631.561

**Published:** 2025-01-29

**Authors:** Stephanie Vergara-Mojica, Alexandra Zodo, Meghan Niego, Mirza Ali, Alfredo J Mena Lora

**Affiliations:** Ross University, Chicago, Illinois; Ross University, Chicago, Illinois; Saint Anthony Hospital, Chicago, Illinois; Saint Anthony Hospital, Chicago, Illinois; University of Illinois Chicago, Chicago, Illinois

## Abstract

**Background:**

High community transmission of respiratory viruses can lead to employee illness, absenteeism, and the risk of spread to both patients and healthcare workers (HCWs). Masking in healthcare settings can mitigate these risks. Our hospital developed a policy to adjust the level of masking required based on current community transmission rates, intensifying or relaxing mandates as necessary. This study aims to evaluate the impact of our tiered masking policy on employee absenteeism.

**Figure:**
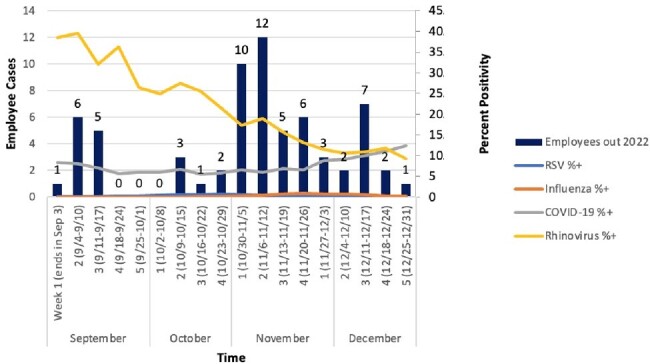

Weekly respiratory viral metrics and employee influenza-like illness for 2022

**Methods:**

Retrospective review of employee health data, public health respiratory virus surveillance metrics, and infection prevention masking requirements at a 151-bed safety-net hospital. Data from September to December 2022, when universal masking was in place, and September to December 2023, when our new masking policy was implemented was compared. In 2023, four masking levels were created: red always requires masks for HCWs and patients; orange requires them for HCWs; yellow requires masks for HCWs during patient contact; both orange and yellow recommends but doesn’t require masks for patients; green recommends but does not require masks for HCW and patients. Weekly public health respiratory surveillance data, masking requirements and employees reporting influenza-like illness (ILI) were tabulated and compared before and after our new masking policy.

**Figure:**
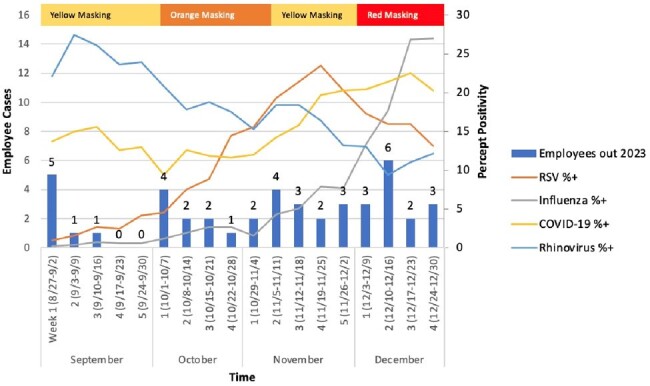

Weekly respiratory viral metrics and employee influenza-like illness for 2023

**Results:**

A total of 66 HCWs reported ILI during the 2022 respiratory viral season compared to 44 in 2023, a 33% reduction. Weekly respiratory viral metrics and HCW ILI is plotted in Figure 1 for 2022 and Figure 2 for 2023. When mask levels were raised to orange on October 6, HCW ILI slightly decreased. Masking changed to yellow on October 22 and correlated with a subsequent increase in ILI. Escalation to red on December 10 was followed by a decline in ILI.

**Conclusion:**

A tiered masking policy in response to fluctuating community transmission appears to be an effective strategy in managing HCW absenteeism due to ILI. Despite higher percent positivity from multiple viruses, a 33% decrease in ILI occurred after implementation of the tiered masking policy, as compared the prior universal masking policy. A tiered masking policy based on percent positivity may be more effective than universal masking.

**Disclosures:**

**All Authors**: No reported disclosures

